# Erratum: Neves KVRN, Machado LMG, Lisboa MN, Steinmann P, Ignotti E. Self-reported clinical history of misdiagnosed leprosy cases in the State of Mato Grosso, Brazil, 2016-2019. Cad Saúde Pública 2023; 39(5):e00279421.

**DOI:** 10.1590/0102-311XER279421

**Published:** 2023-07-17

Onde se lê:

Self-reported clinical history of misdiagnosed leprosy cases in the State of Mato Grosso, Brzil, 2016-2019.

Leia-se:

Self-reported clinical history of misdiagnosed leprosy cases in the State of Mato Grosso, Brazil, 2016-2019.

